# The Impact of COVID-19 on Informal Caregiving and Care Receiving Across Europe During the First Phase of the Pandemic

**DOI:** 10.3389/fpubh.2021.673874

**Published:** 2021-06-16

**Authors:** Michael Bergmann, Melanie Wagner

**Affiliations:** ^1^Munich Center for the Economics of Aging, Max Planck Institute for Social Law and Social Policy, Munich, Germany; ^2^Technical University of Munich, Munich, Germany

**Keywords:** SHARE, COVID-19, informal care, physical and mental health, epidemiological control measures, stay at home orders

## Abstract

**Purpose:** We analyzed the effects of COVID-19 as well as its accompanying epidemiological control measures on health-related outcomes (physical and mental health) and unmet care needs of both caregivers and care recipients across Europe and Israel by taking into account country differences.

**Methods:** We applied comparisons of adjusted predictions, controlling for a large set of relevant respondent characteristics, to investigate changes in the physical and mental health of caregivers and care recipients due to COVID-19. Furthermore, multilevel regression models were used to analyze the effect of individual and contextual indicators on the probability of reporting difficulties in receiving care. For the analyses, we used data from 26 countries with 51,983 respondents over 50 years based on the eighth wave of the Survey of Health, Aging and Retirement in Europe (SHARE), which had to be suspended in March 2020, and the SHARE Corona Survey fielded from June to August 2020.

**Results:** During the first phase of the pandemic in spring/summer 2020, the frequency of providing personal care to parents increased in almost all European countries, while care to children, in turn, decreased. Parental caregivers who increased the frequency of providing personal care reported significantly more mental health strains, that is, feeling sad/depressed and anxious/nervous more often since the outbreak of the pandemic. With respect to receiving care, about one out of five care recipients had difficulty in obtaining adequate care from outside the household during the pandemic. The perception of unmet care needs was significantly associated with country differences regarding the duration of the stay-at-home orders. In contrast, the number of confirmed deaths did not have a significant effect on perceiving difficulties related to receiving care.

**Conclusions:** Our findings show the extent of the burden to which caregivers and care recipients were exposed with respect to the unintended consequences of COVID-19-related epidemiological control measures. There is a great need within this population for interventions, which effectively reduce the burden as well as the symptoms of anxiety or depression for caregivers as well as care recipients. This should be recognized by (health) policymakers and social organizations.

## Introduction

The first phase of the COVID-19 pandemic, which hit European countries at the beginning of 2020, has especially affected those in need of care and those providing the care needed. While media attention has mainly focused on the problematic and often dramatic situation in nursing homes, a major part of care in Europe is provided to people living at home (1–3). This home care is often provided by cohabitating family members and by family members from outside the household (mainly female children) or by paid service providers (4–6). In the context of the COVID-19 pandemic, physical distancing and other epidemiological control measures (e.g., stay-at-home orders, travel restrictions, etc.) instituted in almost all European countries have restricted individuals' access to both formal and informal support resources (7–10). Particularly, older people and individuals with chronic medical conditions have been advised to stay at home as much as possible, raising concerns about the provision of personal care. In addition to these accompanying or *indirect* effects of the pandemic, there are *direct* effects of the virus itself on physical health that might influence the provision of personal care. Thus, it is obvious that caregivers who provide personal care to family members outside their own household are at higher risk of getting infected by COVID-19 themselves, as they regularly travel to and meet with care recipients, accompany them to the doctors and hospitals, and also often do grocery shopping for them. Fearing an infection as well as fearing infecting someone close might therefore also have an impact on the frequency and amount of informal care provision and the use of it (11). Taken together, these direct and indirect effects of the pandemic can be assumed to (a) increase the intensity and burden for caregivers and (b) lead to a worsening of the situation for those who rely on personal care, as less care will be provided, and the remaining amount does not meet the needs of care recipients anymore. In this respect, it is crucial to examine how private care networks have been affected by the pandemic and to what degree personal care could be provided to those who need it most. Furthermore, our knowledge of the possible negative effects of the pandemic on the physical and mental health of care recipients as well as on the caregiving family members is still limited. In particular, we lack reliable and internationally comparable evidence that can increase our knowledge about country differences regarding the challenges caregivers and care recipients are facing during the COVID-19 pandemic as well as with regard to the handling of the pandemic by national governments.

With respect to informal caregiving, we know that personal care is usually done by one main caregiver, who might be supported by other family members and/or by additional formal care providers, the so-called support or care network [e.g., (12, 13)]. During the pandemic, these additional family caregivers often reduced their contacts either voluntarily or forcedly to avoid transmission of the virus and/or to reduce their own risk of infection (14). This has led to smaller care networks and hence to more responsibilities for the main caregiver. Further, many informal caregivers usually receive support from formal care providers, who have often had to close, reduce, or rearrange services since the outbreak of the pandemic (15). In Germany, for example, the provision of ambulant care has been affected by staff shortages (16). In addition, day care and night care centers have had to close (14), and rehabilitation centers and hospitals have sent their patients home in order to free capacity for expected COVID-19 patients (17). Also, many live-in migrant care workers have returned to their home countries during the pandemic and have been unable to cross European borders afterwards, as many work without an official work contract (18, 19). As a consequence, many (single-country) studies have reported a substantial increase in carers helping people outside their own household and in the average time spent on caring (9, 13, 20, 21). Concerning the situation of caregivers, Eggert et al. (14) provided evidence that one out of three caregivers in Germany reported a worsening of the care situation after the outbreak of COVID-19. Evidence from several countries shows that large proportions of caregivers have experienced an increased burden and stress-related symptoms, like trouble sleeping, since the outbreak of the pandemic [e.g., (9, 13, 22, 23)]. Furthermore, informal caregivers frequently reported worsened physical and mental health, such as being depressed or anxious as well as feeling more socially isolated and lonely [e.g., (9, 24, 25)]. Based on these considerations, we formulated the following hypotheses:

H1: COVID-19 and its accompanying control measures lead to an increase in the frequency of providing informal family care to those who rely most strongly on personal care.

H2: COVID-19 and its accompanying control measures lead to a worsening of physical and mental health for informal caregivers.

H3: Caregivers who have increased the frequency of providing personal care suffer more from physical and mental health strains than caregivers who have not increased the frequency of providing personal care.

Compared to caregivers, evidence concerning how the pandemic and its accompanying epidemiological control measures have affected care recipients is rather scarce. For example, we currently still lack comprehensive knowledge about whether care receivers had more unmet (health) care needs during the first phase of the pandemic and what the consequences are thereof. As older care receivers often have the greatest risk to their health from being infected with COVID-19, they typically are in higher need of health care. In addition, it has been long known that meeting older people's care needs is crucial for maintaining their mental and physical well-being (26). Compared with older adults receiving adequate care, those reporting unmet needs face greater challenges. Since the outbreak of the pandemic, it can be assumed that such challenges have greatly increased. While evidence was mixed in the beginning regarding physical and mental health [(20, 27), cf. (28)], more and more studies have recently reported higher rates of depression and greater loneliness since the onset of the pandemic with respect to older adults (29–31), as well as higher levels of stress, anxiety, and depression among people with health problems or dementia (32–34). In this respect, other studies emphasized reduced opportunities for social interaction and made use of examples in which caregivers reported that their relatives with dementia were frustrated as it was difficult for them to understand why they should not go out or had to reduce contacts (25). This coincides with observations that caregivers in many countries reported a worsening of the care situation (9, 32). Therefore, it is likely that such a worsening will also be noticed by care recipients. In this respect, Comas-Herrera et al. (35) presented indications that the quality of social care services to older adults decreased during the early stages of the pandemic. This can partly be attributed to government distancing guidelines and travel restrictions, which also affected care recipients who rely on the provision of care by people from outside their own household. A study of older adults in the UK during the early stages of lockdown found that public health measures disrupted individuals' access to medical care, including accessing medications and the cancellation or delay of doctors' appointments and surgeries (36). Another British study found that around 40% of outpatient and 60% of inpatient care was canceled by the National Health Service in spring 2020 and that 20% of patients canceled their doctor's visits by themselves (37). In contrast, there is also evidence that the majority of caregivers, particularly when providing personal care to people with more advanced health conditions like dementia, maintained their services (23). Thus, it can be assumed as well that care recipients with severe chronic conditions will be prioritized by the health care system as a vulnerable and high-risk group. Finally, epidemiological control measures affected informal caregivers and care recipients alike, but the intensity and the duration of these measures (as well as its perception and adherence) differed across countries and hence might have exhibited effects in varying degrees (38–40). Therefore, it is crucial to relate country-specific conditions to both changes in caregiving behavior and the unmet care needs of care recipients.

H4: Care recipients suffer more from physical and mental health strains due to COVID-19 and its accompanying control measures compared to non-care recipients.

H5: Indicating difficulties in receiving care is positively associated with problems in getting access to medical treatments.

H6: Contextual (country) characteristics affect respondents' perception of difficulties in receiving care.

Against this background, we focus on how both family caregivers and care recipients in Europe experienced and dealt with the situation during the first phase of the COVID-19 pandemic in spring 2020. In this respect, we contribute to the existing literature in several ways: First, we focus on personal caregiving and care receipt to and from outside the household because these two groups are most directly and severely affected by the pandemic. We hence exclude more common forms of help and support (e.g., obtaining necessities like food or help with household repairs) to analyze the direct and indirect effects of COVID-19 for care recipients who rely on personal care and caregivers who provide care to those in need. By this, we are able to derive a comprehensive picture of the impact of the pandemic on informal care rather than one-sidedly focusing on either caregiving or care receiving. Second, our results are based on a large, high-quality survey derived from full probability samples, which included 26 European countries plus Israel. This country-comparative perspective enables us to better understand the effects and consequences of a global pandemic like COVID-19 and hence is superior to studies from single countries. Third, by extending survey data collected after the first phase of the pandemic with panel information collected before the outbreak of the pandemic, we are able to use the full wealth of information on the situation of people 50+ who have been the hardest hit by COVID-19. In particular, we know details of their economic situation and their health conditions that can feed our analyses. This provides us with crucial context information on respondents'/household situations before the outbreak of the pandemic and enables us to thoroughly investigate how COVID-19 has changed the situation of informal caregivers and care recipients and what the consequences are thereof regarding unmet care needs in particular. Finally, our results increase our understanding with regard to what support is needed most by both informal carers and care recipients due to the direct and indirect effects of COVID-19. This is important for finding common responses to the short-, mid-, and long-term consequences of the pandemic by policymakers and social organizations.

The remainder of this paper is organized as follows: In the Materials and Methods section, we describe the data and measures used for the analyses as well as our analysis strategy. Afterwards, we first explore changes in caregiving during the first phase of the pandemic (Caregiving During the Pandemic section) and then focus on care receiving and the problems care recipients faced in receiving the care they needed in spring/summer 2020 (Care Receiving During the Pandemic section). Finally, in the Discussion section, we discuss our findings and their implications.

## Materials and Methods

### Data Source

The following analyses use Wave 8 (release 0) data from the Survey of Health, Aging and Retirement in Europe [SHARE; (41)], which was suspended in March 2020 (42), and the SHARE Corona Survey fielded from June to August 2020 (43), that is, some weeks after the peak of the first COVID-19 phase in most countries. SHARE is a multidisciplinary panel study providing information on health, socioeconomic status, and social and family networks of respondents aged 50 and over. From 2004, data were collected every 2 years in person (Computer-Assisted Personal Interview; CAPI). By its eighth wave, SHARE included 27 European countries plus Israel. While all waves so far have been conducted face-to-face, the SHARE Corona Survey was done by telephone (Computer-Assisted Telephone Interview; CATI) and covered the most important life domains for the target population and asked specific questions about infections and life during the lockdown (44). For most countries, the SHARE Corona Survey was based on the complete national SHARE panel sample, including both panel members who have not been interviewed before the suspension of fieldwork and panel members who have already been interviewed face to face in Wave 8. Only in two countries (the Netherlands and Sweden) needed to have a stratified subsample to be selected due to funding issues. Our analyses were based on data from 51,983 respondents over 50 years in the SHARE Corona Survey (we excluded Austria from our analyses, because fieldwork there only started at the beginning of August when most other countries had nearly finished the SHARE Corona Survey). The preliminary average response rate based on eligible respondents participating in Wave 8 was 79%, ranging from 58% (Luxembourg) to 96% (Romania). There were 18,398 respondents who exclusively answered the SHARE Corona survey through telephone interview after the outbreak of the pandemic but could not be successfully approached in person before the suspension of the Wave 8 fieldwork. These data have been carefully augmented with information from previous waves where appropriate (45–52). The SHARE data are unanimous based on full probability samples (53, 54), providing internationally comparable data that can add important insights to recent studies, which are frequently restricted to the national level. Both the methodological rigor and the cross-country harmonization of SHARE are prerequisites to properly investigate the direct and indirect effects of a global pandemic like COVID-19 and hence support evidence-based policymaking. By further including country-specific data not only on the pandemic itself but also on accompanying epidemiological control measures (39), our results offer a unique perspective that allows to compare how the high-risk group of older respondents coped with the crisis, how the national governments and health care systems responded to the pandemic, and which lessons should be drawn from the variability between countries for the future.

### Measures

#### Caregiving and Care Receiving

In our analyses, we focused on informal (i.e., non-professional, unpaid) caregiving and care receiving, excluding more common forms of help or support [for the latter see, e.g., (55)]. Caregiving was measured by the following question: “Since the outbreak of Corona, did you provide personal care to others outside your home?” followed by a request to indicate the frequency and the recipient of the caregiving activities (if applicable): “How often did you provide personal care to the following people from outside your home compared to before the outbreak of Corona; less often, about the same, or more often?” The list of recipients included one's own children; one's own parents; other relatives; and other non-relatives like neighbors, friends, or colleagues. Care receiving was asked the following way: “Did you regularly receive home care before the outbreak of Corona?” In contrast to caregiving, there were no follow-up questions on the frequency or on the provider of personal care. Instead, we used the respondents' answers on possible difficulties in receiving personal care for our analyses: “Since the outbreak of Corona, did you face more difficulties in getting the amount of home care that you need?” It has to be noted that the use of the term “home care” in the generic version of the SHARE Corona questionnaire potentially complicated distinguishing the receipt of informal and formal care. However, a careful inspection of the different translations did not reveal any systematic differences across countries. Furthermore, our analyses regarding the associations of care receiving were not substantially affected by this issue.

#### COVID-19-Related Health Outcomes

To explore the direct and indirect effects of the pandemic, we included several indicators that measured changes in respondents' physical and mental health since the outbreak of the COVID-19 crisis. In this respect, we used respondents' self-rated health (“If you compare your health with that before the outbreak of Corona, would you say your health has improved, worsened, or stayed about the same?”) as well as indications of depression (“In the last month, have you been sad or depressed?”), anxiety (“In the last month, have you felt nervous, anxious, or on edge?”), sleeping problems (“Have you had trouble sleeping recently?”), and loneliness (“How much of the time do you feel lonely? Often, some of the time, or hardly ever or never?”). We then generated dichotomized variables that indicate a worsening of respondents' self-rated physical and mental health in case respondents confirmed that their health strains have increased since the outbreak of the pandemic (“Has that been more so, less so or about the same as before the outbreak of Corona?”). In addition, we included a measure that indicates whether the respondent was directly affected by COVID-19, using a set of questions on (a) having experienced symptoms, (b) having been tested for COVID-19, and (c) having been hospitalized. For analyzing the associations with care receiving, we further included two dichotomized variables measuring problems regarding a continuation of medical treatments since the outbreak of the pandemic: first, whether a medical treatment was canceled by the respondents themselves because of being afraid of getting infected and second, whether a planned medical treatment was postponed or denied by the doctor or medical facility.

Covariates that could potentially confound the relationship with caregiving and care receiving were selected according to existing knowledge regarding their predictors [e.g., (56–58)] and included the following.

#### Socio-Demographics

We used the respondents' sex (0: male, 1: female) and their age at interview. Further, we coded the level of education attained based on the Internal Standard Classification of Education 1997 (ISCED-97). Respondents were then grouped into three categories [e.g., (59)]: primary education (ISCED-97 score: 0–2), secondary education (ISCED-97 score: 3), and post-secondary education (ISCED-97 score: 4–6).

#### Living Conditions

We used information on the respondents' type of living area (0: rural area, 1: urban area like a large town or big city), household composition (0: living with a partner, 1: living alone), and whether s/he is living in a nursing home. Furthermore, we measured each respondent's economic status by a question that asked the degree to which respondents can make ends meet (0: with great/some difficulty, 1: fairly easily/easily) and included a measure related to whether the respondent was employed (including self-employment) at the beginning of the outbreak of COVID-19.

#### Physical Health Before the Pandemic

To control for respondents' physical health before the pandemic, we used indicators from the previous SHARE waves that have been conducted before the outbreak of COVID-19. In this respect, we used the reversed 5-point scale on respondents' self-rated health (0: poor, 1: fair, 2: good, 3: very good, and 4: excellent). Furthermore, we used three disability measures to assess (a) difficulties in basic activities of daily living [ADL; (60)], such as dressing, walking, bathing/showering, or using the toilet (0: no limitations, 1: ≥1 limitation); (b) difficulties in instrumental activities of daily living [IADL; (61)], such as using a map, preparing a meal, shopping for groceries, or making telephone calls (0: no limitations, 1: ≥1 limitation); and (c) long-standing activity limitations based on the Global Activity Limitation Index [GALI; (62)] that refers to general health problems in activities people usually do (0: not limited, 1: somewhat/severely limited).

#### Governmental Policy Measures

To assess differences in national policy responses to the pandemic, we used the Oxford COVID-19 Government Response Tracker [OxCGRT; (39)] that considers different policy measures (e.g., school and workplace closures, stay-at-home orders, or restrictions on internal movement) and also provides chronological data for each country regarding the cumulative number of infections and confirmed deaths due to COVID-19. Based on these data, we built two indicators that we applied in our multivariate analyses: first, we used the cumulative number of confirmed deaths due to COVID-19 in each country to measure the current severity of the pandemic with respect to older, at-risk respondents. Second, we calculated the duration of stay-at-home orders in days to measure the length and stringency of a specific restriction, which is expected to directly influence the possibilities of caregiving and care receiving. In both cases, we matched the Oxford data to the SHARE Corona Survey data via the specific interview date of all respondents (63). By this, we were able to match precisely the country-specific context information on the pandemic to the respondents' answers on the day of the interview.

### Analytic Strategy

We restricted our analyses to caregiving to and care receiving from someone outside one's own household because, other than personal care within the same household, we expect that care activities outside one's own household were more severely affected by the pandemic and accompanying epidemiological control measures, such as stay-at-home orders. To address our research questions, we first descriptively explored country differences regarding the prevalence of providing and receiving personal care since the outbreak of the COVID-19 crisis. Afterwards, we investigated differences in relevant health-related outcomes between caregivers and care recipients on the one hand and respondents who did not provide care to or receive care from someone outside their own household on the other. Here, we were particularly interested in differences with regard to a worsening of physical and mental health as well as the degree of affectedness by COVID-19. In this respect, we used comparisons of adjusted predictions, controlling for a large set of individual respondent characteristics. In particular, we controlled for the respondents' sex, age, level of education, household composition (i.e., living alone or with a partner), area of living (rural vs. urban), subjective economic status, and whether the respondent was (self-) employed before the pandemic. Furthermore, we controlled for respondents' self-rated health and limitations in ADL and IADL as well as in activities people usually do (GALI) due to long-standing health problems before the pandemic. Finally, country dummies were included to control for regional differences. With this approach, we were able to compare two hypothetical populations (e.g., non-caregivers and caregivers) that have identical values on all independent variables included in the model. The logic is similar to that of a matching study: Because the only difference between the two populations is the provision/receipt of care, caregiving/care receiving can be attributed with much more confidence as the cause of differences in the probabilities of reporting physical and mental health strains [see (64)].

In a second step, we used multilevel regression models with country as the level-two identifier to address the underlying hierarchical structure of the data and to analyze the effect of individual and context indicators, which are expected to play an important role during the pandemic, on the probability of reporting difficulties in receiving personal care. The multilevel approach enables analyzing variables from different levels simultaneously by properly taking into account the statistical dependencies between the observations to adjust standard errors, which are likely to be biased if the hierarchical structure of the data is ignored [e.g., (65–67)]. The dependent variable, difficulties in receiving personal care, was treated as binary in the multilevel model, with the customary logit function defined as logit(*x*) = ln[*x* / (1 – *x*)]. The predicted value for *P*_ij_ in the general logistic multilevel model was extended to include an explanatory variable *X* at the individual level, and a country-level variable *Z* can be written as follows:

$$\begin{array}{l} \text{logit}\left(P_{ij}\right)=\;{\text{γ}}_{00}+{\text{γ}}_{10}{\text{X}}_{\text{ij}}+{\text{γ}}_{01}{\text{Z}}_{\text{j}}+{\text{u}}_{0\text{j}}, \end{array}$$

where the random intercept γ_00_ is shared by all countries, while the residual term *u*_0j_ is specific to country j and assumed to follow a normal distribution with variance $\sigma_{u_{0}}^{2}$. To quantify the extent to which reporting difficulties in receiving care varies between countries, the intraclass correlation coefficient (ICC) was calculated as follows in the intercept-only model without explanatory variables:

$$\begin{array}{l} \text{ICC}=\frac{\sigma_{u_{0}}^{2}}{\sigma_{u_{0}}^{2}+\sigma_{e}^{2}}, \end{array}$$

where $\sigma_{u_{0}}^{2}$ is defined as the country variance at level two, and the individual variance at level one, $\sigma_{e}^{2}$, was fixed to π^2^ / 3 ≈ 3.29 in logistic multilevel regressions [e.g., (67, 68)]. The ICC ranges between 0 and 1. An ICC of 0 indicates that no variance is attributable to country differences, whereas a value of 1 means that all variance is attributable at the country level. Higher values hence indicate a stronger influence of country differences on the respondents perceiving difficulties in receiving care. Because variance components in multilevel logistic regressions cannot be directly compared across models with and without explanatory variables due to the fixed level-one variance, we followed the approach by Hox (67) and calculated a scale correction factor for each model with explanatory variables. With this correction, we were able to assess the amount of variance explained separately at the different levels. As explanatory variables, we included the measures described above, that is, socio-demographics (sex, age, and level of education), living conditions (area type, household composition, living in a nursing home, and subjective economic status), physical health before the pandemic (self-rated health and health limitations), COVID-19-related health outcomes (worsened health, direct affectedness, and mental health strains), and access to medical treatments at the individual level as well as COVID-19-related context effects (confirmed deaths and duration of stay-at-home orders) at the country level. To control for potential sample selection effects regarding the augmentation of respondents' background information, we included a dichotomous variable indicating which respondents could only be interviewed by telephone due to the suspension of regular fieldwork in Wave 8. All variables were standardized with regard to the overall sample mean. Analyses were performed using Stata 14 SE (69) based on robust standard errors and with calibrated weights for the SHARE Corona Survey sample as provided by SHARE. For the multilevel logistic regression model, we use Stata's *melogit* command, which is based on a maximum likelihood estimation procedure using adaptive quadrature with seven integration points.

## Results

### Caregiving During the Pandemic

**Figure 1:**
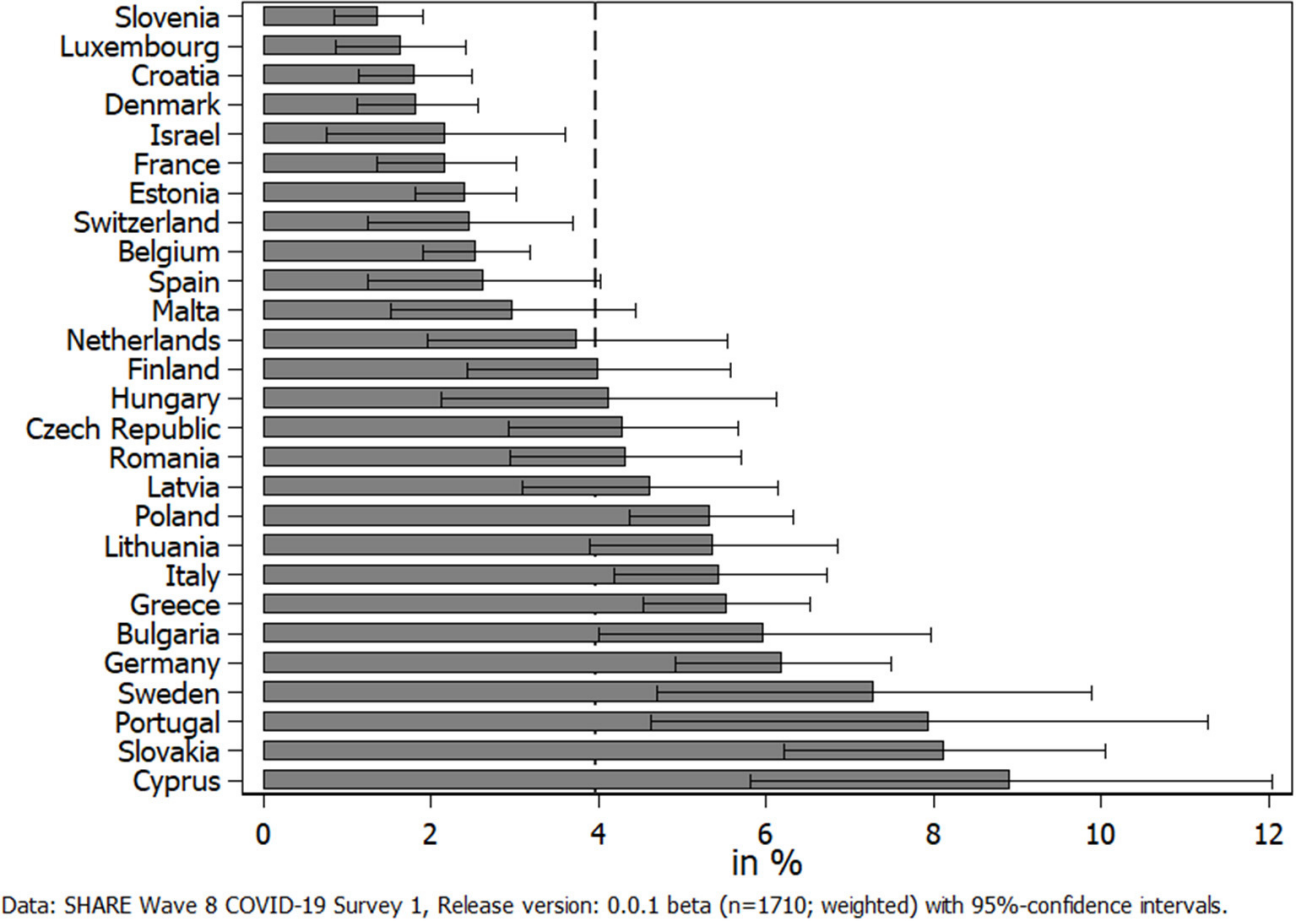
Percent of respondents providing personal care to others outside their own household since the outbreak of the pandemic.

We started our analyses with reporting the overall prevalence of caregiving across Europe during the first phase of the pandemic. On average, 4% of all respondents (*n* = 1,710) indicated that they have provided personal care (excluding general help and support) to someone outside their own household since the outbreak of the pandemic. Figure 1 shows rather large differences between countries. While Slovenia brought up the rear with only 1.4%, in Cyprus, respondents provided care about six times more often (8.9%). In addition, it was noticeable that due to the small sample size, standard errors were quite large in some countries. Further, there was no clear pattern visible with respect to region, and apart from Germany and Sweden, only countries from Southeastern Europe exhibited a prevalence of providing personal care significantly above the average.

**Figure 2:**
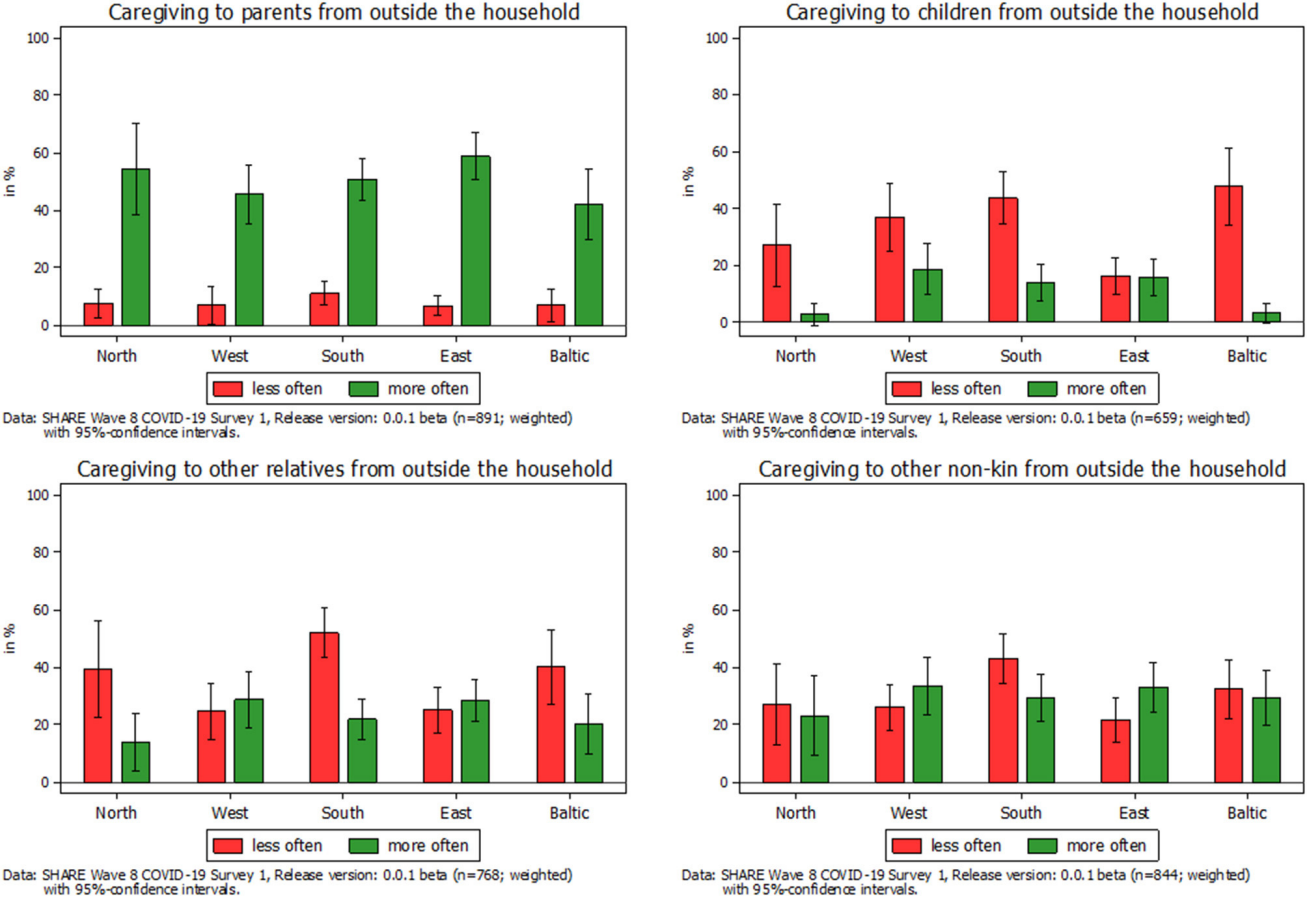
Change in frequency of caregiving to someone outside their own household by type of care relation.

What cannot been seen in Figure 1 is whether the frequency of providing personal care changed due to COVID-19 and whether this differed with respect to the care relationship. In the following, we therefore differentiated between different recipients who received personal care from someone outside their own household when investigating changes in providing care (see Figure 2). Most striking in this respect was the huge increase in children providing care to their parents since the outbreak of the pandemic, which is visible in the upper left graph of Figure 2. This increase was consistent across different regions in Europe, which distinguishes between Northern European States (Sweden, Denmark, and Finland), Western European States (Belgium, France, Germany, Luxembourg, the Netherlands, and Switzerland), Southern European States (Croatia, Cyprus, Greece, Israel, Italy, Malta, Portugal, Slovenia, and Spain), Eastern European States (Bulgaria, Czech Republic, Hungary, Romania, Poland, and Slovakia), and the Baltic States (Estonia, Latvia, and Lithuania): Between 42% (Baltic States) and 59% (Eastern Europe) of all parental caregivers declared that they had increased the provision of personal care to their parents since the outbreak of the pandemic, that is, on average, more than every second parental caregiver reported an increase. In contrast, only between 7 and 11% indicated that they had decreased the personal care given to their parents. The rest, on average about 40%, had neither increased nor decreased their caregiving activities to parents since the outbreak of the pandemic. The picture considerably changed when looking at parents who provided personal care to their children (see upper right graph of Figure 2). Here, about one third of all caregivers providing personal care to their children reported a decrease, while only about 12% reported an increase. Thus, with the exception of the Eastern European States, decreases in the provision of care from parents to their children clearly outweighed the increases. Finally, with respect to other relatives and other non-kin, our findings were more balanced (see lower two graphs of Figure 2). In both cases, the overall share of caregivers who had decreased their respective caregiving activities was larger than the share who indicated an increase (38 vs. 24% with respect to other relatives and 32 vs. 30% with respect to other non-kin). This was mainly due to the countries in Southern Europe, where decreases most clearly outweighed increases in providing personal care.

Based on these findings, we were interested in two things: (a) whether caregiving in general was associated with higher physical and mental health strains compared to non-caregivers and (b) whether the strongly increased personal care activities of children to their parents in particular were associated with higher physical and mental health strains compared to respondents who had not increased their parental caregiving activities. To answer these questions, we first compared all caregivers with all non-caregivers in our sample (columns 2 and 3 in Table 1), while controlling for a broad range of relevant individual characteristics including health conditions that are well-known to differ between caregivers and non-caregivers [e.g., (57, 58)] and otherwise might have biased our results. Table 1 thus presents adjusted predictions that are controlled for the covariates presented in the Measures section. With this approach, we were able to compare two hypothetical populations (e.g., non-caregivers and caregivers) that have identical values on all independent variables included in the model.

**Table 1:** Adjusted predictions of health-related outcomes by caregiving and changes in parental caregiving.

	**Non-caregivers (%)**	**Caregivers (%)**	**Parental caregivers, personal care → / ↓ (%)**	**Parental caregivers, personal care ↑ (%)**
Worsened health	8	7	6	6
Affected by COVID-19	7	9	13	13
Felt sad/depressed more often	16	19^**^	13	27^***^
Felt anxious/nervous more often	21	26^**^	21	36^***^
Had trouble sleeping more often	8	10	10	12
Felt lonely more often	12	13	11	12
*N*	49,969	1,710	439	452

*Data: SHARE Wave 8 COVID-19 Survey 1, release version: 0.0.1 beta and SHARE Wave 8, release version: 0 (weighted). Entries are adjusted predictions, controlling for sex; age; level of education; household composition; area of living; economic status; (self-) employment; self-rated health; ADL, IADL, and GALI before the pandemic; and respondent's country. Significance level:*

* *p < 0.05*,

** *p < 0.01*,

*** *p < 0.001 [significances based on average marginal effects (AMEs) refer to respective previous column]*.

When comparing the entries of column 2 (labeled “Non-caregivers”) with those of column 3 (“Caregivers”), we can see that caregivers, on average, indicated more mental health strains compared to non-caregivers. Since the outbreak of the pandemic, caregivers have felt depressed or sad significantly more often (+3 percentage points) and in particular anxious or nervous more often (+5 percentage points) than non-caregivers. In addition, they slightly more often struggled with sleeping problems (not significant) but did not feel lonely more often. Further, general physical health seemed to be unaffected since the outbreak of the pandemic. Thus, the share of caregivers who indicated a worsening in general health was very similar to the share of non-caregivers. In this respect, it has to be stated that the overall prevalence of respondents indicating a worsening in health was low (about 7–8%, respectively). Finally, with regard to the direct effects of COVID-19 on health, it can be seen that only slightly (not significantly) more caregivers reported that they had been personally affected by the virus compared to non-caregivers. However, any further developments should be followed closely because an increase in the affectedness of caregivers might have strong implications for those who rely on the provision of personal care and at the same time are among the highest risk group (6).

When exploring whether parental caregivers who increased their provision of personal care differed from parental caregivers who did not increase (i.e., either decrease or maintain) the frequency of providing personal care to their parents, we see similar patterns: while general physical health and direct affectedness by COVID-19 again did not differ much, parental caregivers who increased the frequency of providing personal care reported many more mental health strains. The differences were most pronounced with respect to feeling sad/depressed and anxious/nervous more often since the outbreak of the pandemic: more than twice as many parental caregivers who reported an increase of their care activities indicated that they had felt sad or depressed more often since the outbreak of the pandemic, compared to those parental caregivers with the same amount or a decrease in their caregiving activities (+15 percentage points or nearly one out of three). With respect to feeling anxious or nervous more often, the difference was also substantial. Here, about 36% of parental caregivers with an increase in personal care indicated that they had felt anxious or nervous more often, compared to only 21% of parental caregivers who did not increase their caregiving activities. With regard to sleeping problems and feeling lonely more often, the differences were much smaller and not significant.

**Figure 3:**
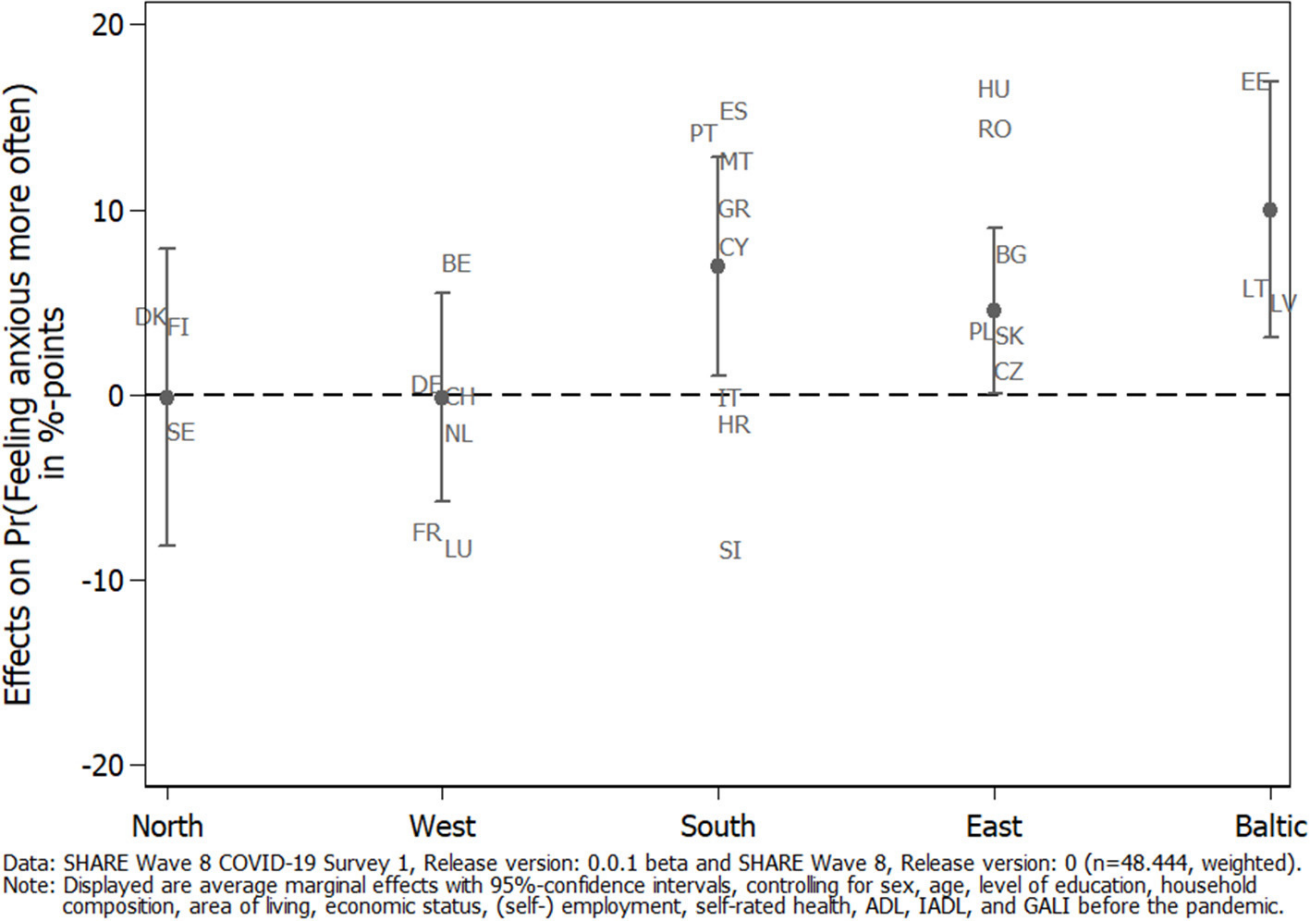
Average marginal effects (AMEs) of caregiving on the adjusted prediction of feeling anxious/nervous more often since the outbreak of the pandemic by geographical regions.

To investigate country differences, we calculated the country-specific average marginal effects of caregiving in general on the adjusted predictions of feeling anxious or nervous more often since the outbreak of the pandemic. Figure 3 shows that caregivers in Southern European countries had a significantly higher probability of reporting anxiety more often compared to non-caregivers, with Spain, Portugal, and Malta as the countries with the highest probabilities. The same is true for the Baltic States, in which caregivers from Estonia reported anxiety most often. Eastern European countries were also slightly above a significant level, and no effect could be found in Northern and Western European countries. This illustrated that there indeed were country differences with regard to effects of the pandemic on caregivers' mental health, which should be taken into account.

### Care Receiving During the Pandemic

**Figure 4:**
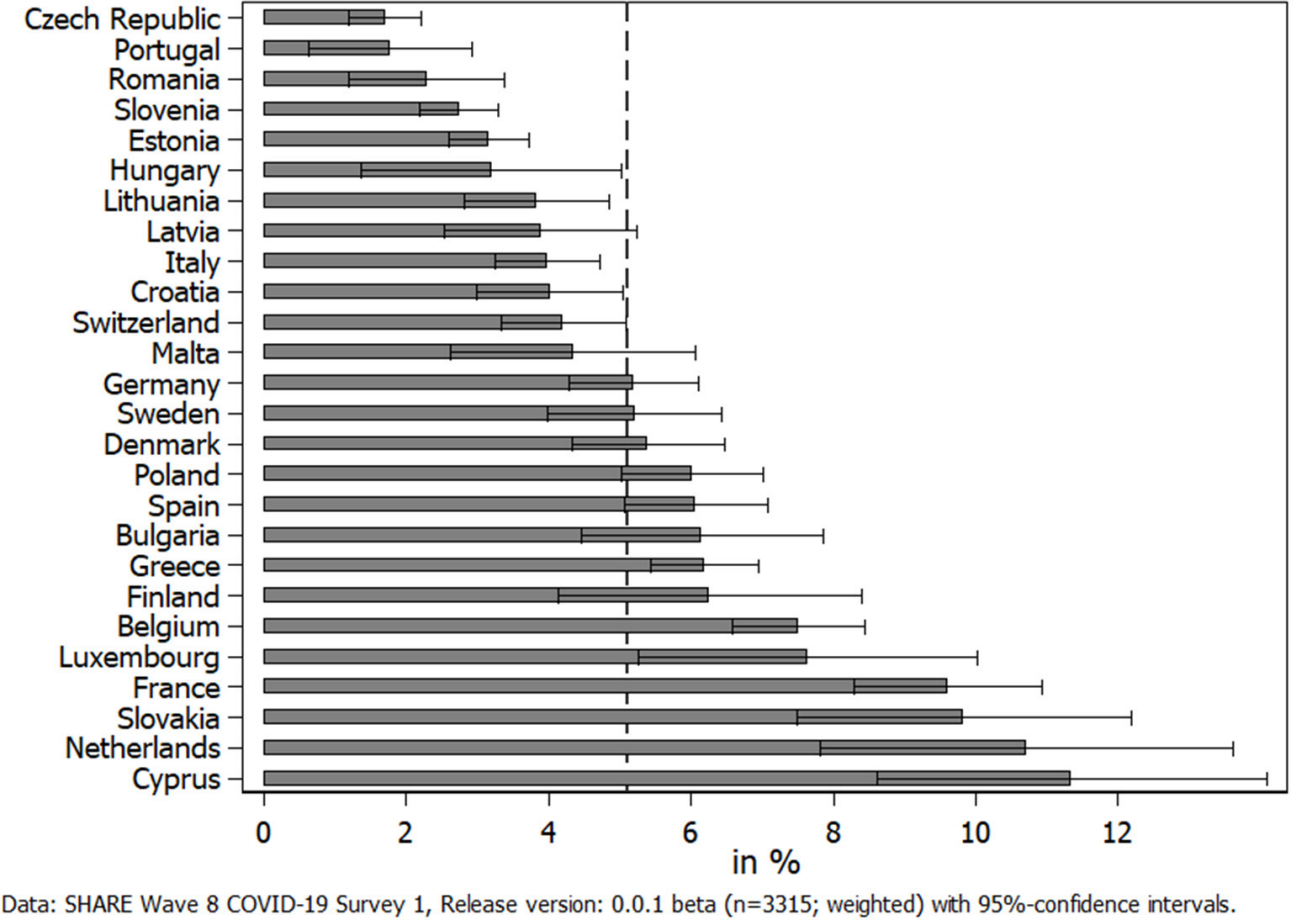
Percent of respondents receiving home care by others from outside their own household since the outbreak of the pandemic.

When turning to care recipients, we first looked at the prevalence of care receiving across countries participating in SHARE (see Figure 4). Overall, about 5% of all respondents in our sample received home care (*n* = 3,315; Israel was excluded from this overview due to a potential mix-up between formal and informal home care activities). Figure 4 again shows large differences between countries. The Czech Republic had the lowest number of care recipients (about 2%), while again Cyprus was the frontrunner with more than 11%. When geographically grouping countries, it was noticeable that Western European countries exhibited a larger share of care recipients. While it can be argued that the age distribution in the national samples affected the countries' ordering, this explanation could be ruled out. Thus, the Czech Republic and Portugal both had rather old samples (69 and 70 years, respectively) but at the same time showed the lowest percentage of care recipients. In addition, Slovakia and Cyprus had much younger samples (between 65 and 66 years), but both exhibited a much higher share of respondents receiving care.

**Figure 5:**
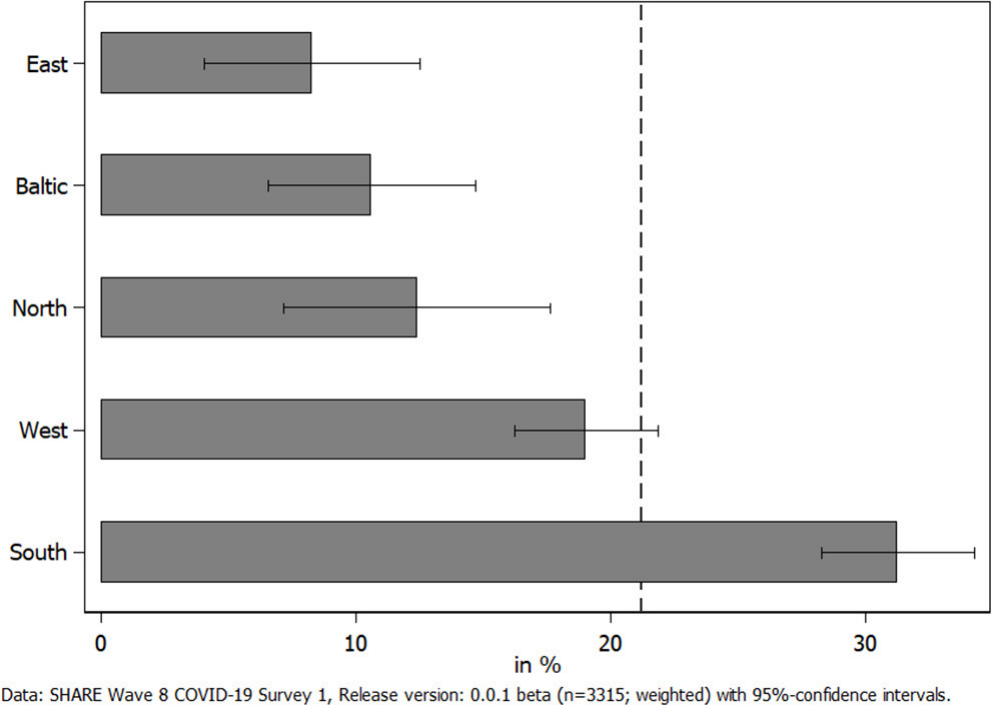
Percent of care recipients facing difficulties in receiving home care since the outbreak of the pandemic.

Next, we investigated how the utilization of (home) care was perceived by those receiving care to answer the question if COVID-19 negatively affected the receipt of personal care in Europe. In this respect, Figure 5 shows the share of care recipients who reported that they faced difficulties in receiving care by geographical regions. We did not differentiate between countries here, as the sample size for receiving home care in some countries was very low and might have jeopardized results. Overall, about 21% of all care recipients reported difficulties in receiving care. This share was by far the highest in Southern European countries: More than every third care recipient in these countries reported difficulties in receiving care since the outbreak of the pandemic, while it was <1out of 10 in Eastern Europe.

Based on these findings, we were further interested in whether care receiving in general during the pandemic as well as the perception of difficulties therein were associated with physical and mental health problems or with restrictions in the health care system (i.e., accessing medical treatments). To answer this question, we first compared all care recipients with all non-care recipients in our sample, while again controlling for relevant individual characteristics including health conditions and country dummies. Table 2 reveals that care receiving actually was associated with worsened health: compared to non-care recipients, care recipients indicated a significantly worsened general physical health (+2 percentage points). In addition, significantly more care recipients (+3 percentage points) reported that they personally had been affected by the virus (i.e., having had symptoms, having been tested, or having been hospitalized). The same was true for most of the indicators regarding mental health strains: care recipients significantly more often reported that they felt sad/depressed, anxious/nervous, and lonely (about +2 percentage points, respectively). With regard to sleeping problems, there was no significant difference. The same was the case for respondents' access to appropriate medical treatment: treatments and appointments had not been canceled more often by care recipients themselves or by medical facilities than with respect to non-care recipients.

**Table 2:** Adjusted predictions of health-related outcomes by care receiving and difficulties in receiving care.

	**Non-care recipients (%)**	**Care recipients (%)**	**Care recipients without difficulties in receiving care (%)**	**Care-recipients with difficulties in receiving care (%)**
**Physical and mental health**				
Worsened health	8	10^*^	18	21
Affected by COVID-19	7	10^**^	8	8
Felt sad/depressed more often	16	18^*^	23	26
Felt anxious/nervous more often	21	24^*^	24	29^*^
Had trouble sleeping more often	8	9	11	12
Felt lonely more often	12	13^*^	20	24
**Access to medical treatments**				
Medical treatment canceled by respondent	12	12	13	20^**^
Medical treatment postponed/denied	28	27	29	32
*N*	48,364	3,315	2,588	707

*Data: SHARE Wave 8 COVID-19 Survey 1, release version: 0.0.1 beta and SHARE Wave 8, release version: 0 (weighted). Entries are adjusted predictions, controlling for sex; age; level of education; household composition; area of living; economic status; self-rated health; ADL, IADL, and GALI before the pandemic; and respondent's country. Significance level:*

* *p < 0.05*,

** *p < 0.01*,

*** *p < 0.001 [significances based on average marginal effects (AMEs) refer to respective previous column]*.

When turning to differences between care recipients with and without perceived problems in receiving care, the picture was somewhat different: now, general health no longer differed significantly between the two comparison groups, that is, worsened physical health was not significantly correlated with indicating difficulties in receiving care, although the absolute difference was even slightly larger than before (+3 percentage points). Mental health strains, at least partly, were still related to perceiving difficulties in care receiving: those care recipients who reported difficulties in receiving care felt anxious or nervous significantly more often (+5 percentage points) compared to care recipients who did not. Further, care recipients who reported difficulties in receiving care more often felt sad/depressed and lonely and had sleeping problems more frequently compared to care recipients who did not have such difficulties. However, none of these indicators reached a significant level. In contrast, care recipients who indicated difficulties in receiving care significantly more often reported that they canceled a medical treatment by themselves (+6 percentage points). Finally, there was no significant difference between care recipients who indicated or did not indicate difficulties in receiving care with regard to medical treatments being postponed or denied by a doctor or medical facility.

**Figure 6:**
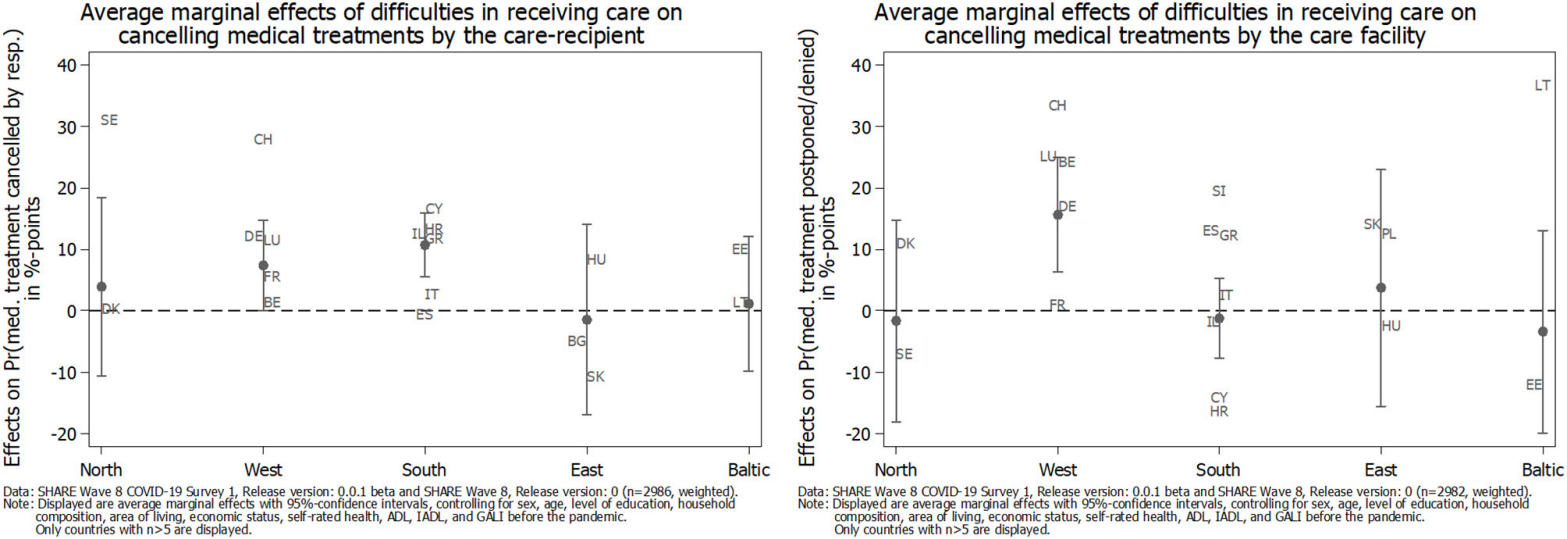
Average marginal effects (AMEs) of difficulties in receiving care on the adjusted prediction of canceling medical treatments since the outbreak of the pandemic by geographical regions.

To explore whether these latter differences varied across countries, we calculated country-specific average marginal effects of the difficulties in receiving care (see Figure 6), both for reporting medical treatments that had been canceled by the care recipients themselves (left graph) and those that have been postponed or denied by medical facilities instead (right graph). In this respect, the left part of Figure 6 shows that the significant difference of canceling medical treatments by care recipients themselves, which has been reported in Table 2, was mainly driven by Southern European (and to a lesser degree by Western European) countries that had been hit rather hard by the first phase of the pandemic. Additionally, the share of care recipients who had a medical appointment canceled by their doctor or medical facility was highest in Western European countries (see right part of Figure 6). This confirmed, for example, the situation in Switzerland or Belgium, where care professionals have been advised to prioritize their care and to assess whether the care is essential or can be postponed (15). Although the standard errors for these effects were rather high, our findings illustrate that the COVID-19 pandemic had different direct and indirect consequences for care recipients across Europe, dependent on the interaction between the severity of the pandemic and the (technical and personal) equipment of the national health care system.

In the last step, we analyzed the determinants of perceiving difficulties in receiving care. For this, we used a multilevel logistic regression model to account for country differences that might influence respondents' answers. First, our analysis revealed that indicating difficulties in receiving care differed significantly between countries. This was reflected in the ICC of the intercept-only model, which was 0.888/(3.290 + 0.888) = 21.3%, that is, about one fifth of the total variance in perceiving difficulties in receiving care was attributable to differences between countries. The intercept-only model also gives us a benchmark value of the deviance (i.e., the degree of misfit of the model), which can be used to compare models with additional explanatory variables. From Table 3, it can be concluded that the deviance went down when including explanatory variables at the different levels, thus indicating an improved model fit. A formal chi-square test to evaluate the difference of the deviances indicated significant improvements of the model fit when including all level-one and level-two predictors, respectively. To further analyze how much residual error is left at the distinct levels and to assess the amount of explained variance at the different levels in multilevel logistic regressions, we needed to bring the sequential models to the same scale [see (67)]. Table 3 presents the rescaled variances from our multilevel logistic regression models (the full model with all parameter estimates can be found in Table A1 in the Supplementary Material). We see that after including respondent characteristics at the individual level (level-one predictors) and context characteristics at the country level (level-two predictors), the residual error variance at the country level decreased compared to the intercept-only model. We can interpret the respective differences as the amount of variance explained by introducing explanatory variables at the different levels: the rescaled explained variance at the country level was about 10% after including individual characteristics and about 36% after including individual and country characteristics. This result showed that the amount of variance explained by respondent characteristics at the country level was rather small, which reflects the fact that the included level-one explanatory variables were distributed more or less equally across countries. Adding the country-level explanatory variables (i.e., confirmed deaths and duration of stay-at-home orders) did not change the residual variance at the first level because the second-level variables cannot predict individual-level variation. However, the country-level residual variance went down to 0.570, which translated into 35.8% of the explained variance at the country level by both respondent and country predictors. Most of the predictive power of the model was hence attributable to context predictors that differed across countries.

**Table 3:** Rescaled estimates of individual ($\sigma_{R}^{2}$) and country residual variance ($\sigma_{u_{0}}^{2})$ of sequential random intercept models regarding respondents' answers on difficulties in receiving care.

	**Intercept-only**	**Random intercept with level-1 predictors**	**Random intercept with level-2 predictors**
$\sigma_{R}^{2}$	3.290	3.084	3.084
$\sigma_{u_{0}}^{2}$	0.888 (0.298)	0.796	0.570
Explained $\sigma_{u_{2}}^{2}$ (%)	–	10.3	35.8
Deviance	2,978.1	2,855.2	2,842.9
*X*^2^	280.6^***^	122.9^***^	135.3^***^

Data: SHARE Wave 8 COVID-19 Survey 1, release version: 0.0.1 beta; SHARE Wave 8, release version: 0; and Oxford COVID-19 Government Response Tracker (n = 3092, weighted). Entries are residual variances with standard errors in parentheses for the intercept-only model. The scale correction factor for the variances was 0.937 in models with explanatory variables. Deviance was defined as −2^*^ln (likelihood) with the difference of the deviances following a chi-square distribution. Significance level:

* *p < 0.05*,

** *p < 0.01*,

*** *p < 0.001*.

**Figure 7:**
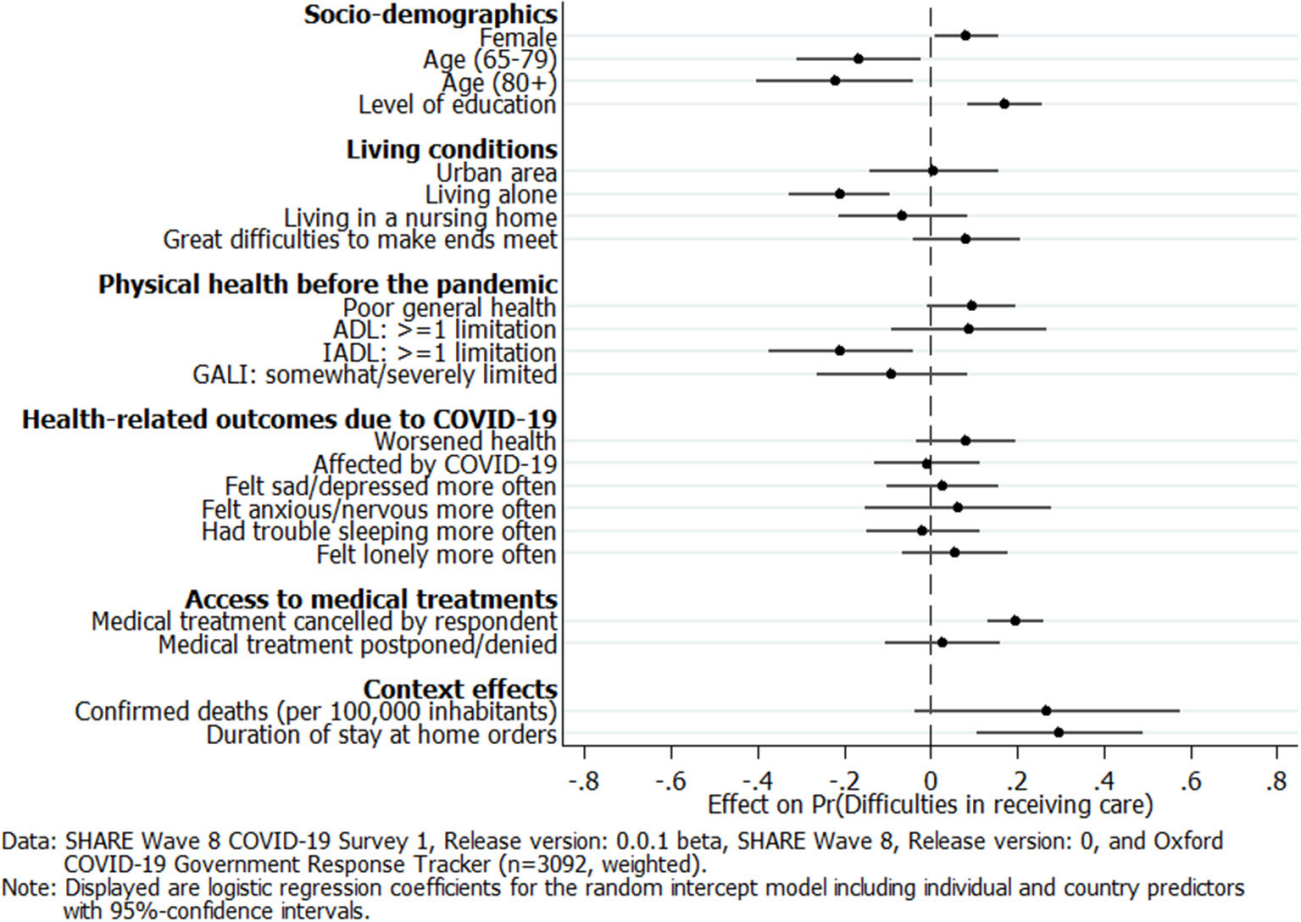
Multilevel logistic regression coefficients of respondent and country predictors on perceived difficulties in receiving care.

Figure 7 graphically presents the coefficients of the respondent- and country-level predictors for the multilevel logistic regression model. We see that female and better-educated care recipients had a significantly higher probability of perceiving difficulties in receiving care since the outbreak of the pandemic. In contrast, older care recipients above 65 years of age had a significantly lower probability of perceiving difficulties in receiving care, compared to younger care recipients below 65 years of age (the reference category). Also, living alone significantly reduced the probability of indicating difficulties in receiving care. Care recipients who indicated great difficulties in making ends meet already before the pandemic as well as those with poor physical health and limitations in basic ADL, such as dressing or showering, tended to express difficulties in receiving care more frequently, although the effect was not significant at the 5% level, respectively. On the other hand, care recipients with limitations in IADL, such as shopping or making phone calls, had a significantly lower probability of indicating that they had unmet care needs since the outbreak of the pandemic. Furthermore, physical and mental health changes during the pandemic were not significantly associated with perceiving difficulties in receiving care. Regarding access to medical treatments during the pandemic, Figure 7 shows that care recipients who canceled medical treatments by themselves for fear of a COVID-19 infection significantly more often indicated unmet care needs, while this was not true for care recipients who had a medical appointment postponed or denied by a care facility. With respect to the country-level predictors, it was evident that more confirmed deaths in a country since the outbreak of COVID-19 until the interview—although increasing the probability of perceiving difficulties in receiving care—were not significantly associated with the outcome variable. In contrast, care recipients from countries in which stay-at-home orders had been implemented for a longer period before the interview had a significantly higher probability of perceiving difficulties in receiving care.

## Discussion

Informal caregivers as well as care receivers have both been hit hard by the outbreak of COVID-19. The pandemic has drastically increased many of the inherent problems of national health care systems in general and of long-term care in particular (70). The spread of the virus together with further COVID-19-related epidemiological control measures have affected the lives of those providing care to others as well as those receiving care from people outside their own household to an unprecedented extent. Against this background, we have focused in this paper on how caregivers and care recipients living at home (the non-institutionalized) have dealt with the situation across Europe. By applying adjusted predictions that controlled for a broad range of relevant respondent characteristics, we were able to present reliable results regarding the association between caregiving and care receiving on the one hand and changes in physical and mental health due to the direct and indirect effects of the pandemic on the other. In addition, our findings, based on a multilevel logistic regression model including explanatory variables at the individual and the country level, helped to answer the questions concerning which care recipients did not get adequate care during the pandemic and how countries differed in this respect.

With respect to informal caregiving, our findings first showed that COVID-19 had a substantial impact on private care networks of caregivers and the persons to whom care was being given. During the first phase of the pandemic in spring 2020, the provision of personal care to parents outside one's own household strongly increased across Europe, thus confirming hypothesis H1, while it decreased for other relatives and non-kin and in particular for children. One reason for the increase regarding parents in need for care was the reduced availability of paid services and care support due to COVID-19-related epidemiological control measures that had to be compensated for by family care. The strong decrease for children on the other hand can be seen as the reverse of the same coin and indicates a strong shift in informal care from the younger to the older generation, which is, on average, more vulnerable and more strongly reliant on informal care from their children rather than the other way round. In addition, our findings clearly showed that caregivers (compared to non-caregivers) more often felt depressed and anxious as a consequence of the pandemic and its accompanying epidemiological control measures, which is in accordance with hypothesis H2 regarding mental health. This finding was even more pronounced for parental caregivers who had increased their caregiving activities since the outbreak of the pandemic. In this population, nearly 30% of parental caregivers indicated feelings of depression more frequently and nearly 40% indicated feeling anxious. Both values were roughly twice as high compared to those for caregivers who did not increase their care activities toward their parents. These numbers strongly confirm hypothesis H3 and at the same time give cause for concern. It clearly shows the extent of the burden to which caregivers were exposed with respect to the unintended effects of the epidemiological control measures and, at the same time, suggests a great need within this population for interventions to effectively reduce the burden as well as symptoms of anxiety or depression. This holds true in particular for Southern European countries, for which we found the strongest negative effects. In contrast, caregivers' physical health remained rather stable during the period of investigation. From this, one could conclude that the direct effects of the virus itself on physical health were less pronounced for caregivers, thus contradicting hypotheses H2 and H3 regarding physical health. Whether this observation will still hold true in the long run and with further waves of the pandemic has yet to be seen and should be monitored closely. In any case, our findings point out that caregivers need compensation for the burden of providing care during the pandemic. Currently, however, they are often expected to protect even more carefully those who rely on their help. Social organizations have long called for improvements to the caregivers' situation, including an actual increase in both their reputation and their payment. Based on our findings, this now seems more reasonable than ever and should be recognized by (health) policymakers, too.

With respect to care receiving, our results showed that the pandemic also negatively affected the health of (home) care recipients. In particular, care recipients (compared to non-care recipients) rated their general physical health significantly worse and felt significantly more depressed, anxious, and lonely, which is in line with hypothesis H4. However, the differences in the adjusted predictions were smaller in absolute size than for caregivers. In addition, care recipients, overall, did not indicate a worsening of their situation with respect to pursuing planned medical treatments compared to non-care recipients. When differentiating between care recipients with and without difficulties in receiving the care they need, we saw that those perceiving difficulties reported substantially more cancellations of medical treatments by themselves due to their fear of a COVID-19 infection. The difference regarding postponements and cancellations by medical facilities between those care recipients indicating difficulties vs. those not indicating difficulties in receiving care was smaller and not significant. Thus, hypothesis H5 is only partly confirmed. This result suggests that the reporting of difficulties in receiving care was more strongly related to subjectively fearing an infection in connection with a medical treatment than objective shortages in the health care system, even though only a small proportion of respondents had actually been infected with COVID-19. This finding, however, varied across countries, with higher shares of care recipients canceling medical treatments by themselves in Southern European countries, which had been affected more in the first phase of the pandemic. In Western European countries, higher shares of care recipients had a medical treatment postponed or denied by their doctor or a medical facility, most likely due to shortages in the national health care system. Independently of its cause, it has to be seen whether canceling necessary medical and therapeutic treatments during the first phase of the pandemic will result in negative long-term consequences on health—and if yes, to what degree. Further, it is noteworthy that, overall, one out of five care recipients reported difficulties in receiving the care they need. In Southern and several Western European countries, which had been hardest hit with respect to the number of confirmed deaths due to COVID-19 in the first phase of the pandemic, this number was even higher. On average, these care recipients also reported slightly more physical and mental health strains with a significantly higher level of anxiety as the most explicit result. This corresponds with our findings regarding caregivers. Hence, it seems that the first COVID-19 phase in spring 2020 can best be characterized by an increase in anxiety for both caregivers and care recipients. However, there is concern that indications of depression will also further increase the longer epidemiological control measures like stay-at-home orders persist (71).

When focusing more closely on the determinants of why care recipients perceived difficulties in receiving care, our results revealed that, in particular, female and more highly educated care recipients, as well as those who canceled their medical treatments by themselves for fear of an infection, had a significantly higher probability of indicating unmet care needs. In contrast, care recipients who were 65 years and older, lived alone, and already suffered from limitations in IADL (e.g., dressing or making phone calls) before the outbreak of the pandemic had a significantly lower probability of perceiving difficulties in receiving care. This indicates that those care recipients who strongly rely on personal care (oldest old, living alone) still received the care they needed during the first phase of the pandemic. In addition, our previous findings with regard to having access to medical treatments also hold in the multilevel setting: care recipients who canceled their medical treatments by themselves more frequently perceived difficulties in receiving care, while medical treatments postponed or denied by care facilities were not significantly associated with a higher probability of unmet care needs. This points out that respondents' subjective fear of a COVID-19 infection outweighed the objective problems of care facilities with respect to the association between getting access to medical treatments on the one hand and indicating unmet care needs on the other. Besides analyzing individual predictors, our analyses also allowed us to include country-specific determinants of the pandemic. Overall, differences across countries with respect to the severity of the pandemic as well as governmental control measures to mitigate COVID-19 indeed helped to explain a substantial part of the country disparities regarding the prevalence of unmet care needs, which is in accordance with hypothesis H6. Our results further revealed that the indirect effects of epidemiological control measures accompanying COVID-19, measured by the length and stringency of stay-at-home orders, turned out to be more impactful in the first phase of the pandemic than the direct effects of COVID-19, measured by the cumulative number of confirmed deaths due to the virus. The longer the stay-at-home orders had already been in place in a country, the higher the probability was of perceiving difficulties in receiving care. This is an important finding that confirms recent studies on the negative consequences of epidemiological control measures in particular for those people who are in need of personal care.

The main limitations of this study are the rather low numbers of caregivers, and even more severe is the number of care recipients who, at the same time, are in presumably good health, which allowed them to participate in the survey. We tried to circumvent this problem by geographically grouping countries to measure the varying effects of the pandemic on caregivers and care recipients across Europe. However, we are aware that more detailed typologies are needed to capture the institutional and cultural differences and also the different government responses to the COVID-19 crisis in order to fully explain the consequences of this global pandemic on caregiving and care receiving. Furthermore, with the data at hand, we lack a comprehensive understanding on the underlying causes of why mental health declined for caregivers as well as for those who intensified their caregiving activities during the first phase of the pandemic: was it the mere burden of caregiving in an unprecedented situation, in which increased care needs and reduced availability of paid services and informal support had to be compensated for by informal family care? Or have worries about care-dependent relatives been the main driver for the strong increase in mental health strains? More research is needed here that also picks up recent findings regarding the interplay between these factors. For example, Kumagai et al. (72) showed that long sleep time was an important risk factor for the recurrence of depression. However, with the current data, it was not possible singling out specific sleeping times. In addition, future research should also explore the interaction of these explanations with the severity of the pandemic, which differs between countries and hence is expected to exhibit different consequences. In this respect, our study was only a first step in answering some of these questions. Others remain, for example, possible selection effects underlying the country differences found regarding a canceling of medical treatments by care recipients themselves. Future research might investigate reasons for these differences more deeply. Finally, we should consider that our findings refer to spring/summer 2020, the first COVID-19 phase after the outbreak. The changing experience with COVID-19 and also the changed mindset with regard to how we now look at the pandemic make it more difficult to evaluate the results against the background of the first COVID-19 phase. Although the current situation is similar in some respects, it differs a lot with respect to the overall perception of the crisis as well as the long-lasting epidemiological control measures and restrictions (“lockdown fatigue”). Therefore, further waves of the pandemic are expected to put even more pressure on the persons under investigation. In this respect, it will be extremely valuable to compare our results with data from a second SHARE Corona Survey, which is actually planned for early summer 2021. This will provide valuable information to evaluate more comprehensively the consequences of COVID-19 across Europe.

## Data Availability Statement

The datasets presented in this study can be found in online repositories. The names of the repository/repositories and accession number(s) can be found at: http://www.share-project.org/data-documentation/share-data-releases.html. Each wave and each release is assigned a persistent DOI. In our article we use SHARE data from Waves 1, 2, 3, 4, 5, 6, 7, and 8 (DOIs: 10.6103/SHARE.w1.710, 10.6103/SHARE.w2.710, 10.6103/SHARE.w3.710, 10.6103/SHARE.w4.710, 10.6103/SHARE.w5.710, 10.6103/SHARE.w6.710, 10.6103/SHARE.w7.711, 10.6103/SHARE.wXcvr.710, 10.6103/SHARE.w8cabeta.001) that are fully available without restrictions to all scientific users world-wide after individual registration (http://www.share-project.org/data-access/user-registration.html).

## Ethics Statement

The studies involving human participants were reviewed and approved by the Ethics Committee of the University of Mannheim (until Wave 4) and the Ethics Council of the Max Planck Society. The patients/participants provided their written informed consent to participate in this study. Written informed consent was obtained from the individual(s) for the publication of any potentially identifiable images or data included in this article.

## Author Contributions

MB and MW contributed equally to conceptualization, methodology, and validation. MB conducted data curation, analyzed the data, and drafted the manuscript. MW reviewed the manuscript. Both authors contributed to the article and approved the submitted version.

## Conflict of Interest

The authors declare that the research was conducted in the absence of any commercial or financial relationships that could be construed as a potential conflict of interest.
