# Supplementary material for: The Impact of COVID-19 on Informal Caregiving and Care Receiving Across Europe During the First Phase of the Pandemic
Source: Front Public Health. 2021 Jun 16;9:673874. doi: 10.3389/fpubh.2021.673874 (PMC8242257; doi:10.3389/fpubh.2021.673874)
Supplement: Supplementary file 1 [file Table_1.DOCX]

**Supplementary Material**

Table A1: Multilevel logistic models for perceiving difficulties in receiving care

|  | Intercept-only model | Random intercept model with individual (level 1) predictors | Random intercept model with country (level 2) predictors |
| --- | --- | --- | --- |
| Intercept | -1.837^***^ | -1.886^***^ | -1.823^***^ |
|  | (-9.11) | (-9.27) | (-10.18) |
| *Socio-demographics* |  |  |  |
| Female |  | 0.085^*^ | 0.083^*^ |
|  |  | (2.26) | (2.24) |
| Age (50-64 years) | Reference category | | |
| Age (65-79 years) |  | -0.173^*^ | -0.166^*^ |
|  |  | (-2.25) | (-2.26) |
| Age (80+) |  | -0.231^*^ | -0.221^*^ |
|  |  | (-2.36) | (-2.38) |
| Level of education |  | 0.163^***^ | 0.170^***^ |
|  |  | (3.90) | (3.91) |
| *Living conditions* |  |  |  |
| Urban area |  | 0.007 | 0.007 |
|  |  | (0.10) | (0.09) |
| Living alone |  | -0.206^***^ | -0.211^***^ |
|  |  | (-3.51) | (-3.59) |
| Living in a nursing home |  | -0.058 | -0.064 |
|  |  | (-0.75) | (-0.84) |
| Great difficulties to make ends meet |  | 0.080 | 0.083 |
|  |  | (1.30) | (1.33) |
| *Physical health before the pandemic* |  |  |  |
| Poor general health |  | 0.092 | 0.094 |
|  |  | (1.76) | (1.80) |
| ADL: >=1 limitation |  | 0.086 | 0.088 |
|  |  | (0.93) | (0.96) |
| IADL: >=1 limitation |  | -0.208^*^ | -0.208^*^ |
|  |  | (-2.45) | (-2.44) |
| GALI: somewhat/severely limited |  | -0.084 | -0.090 |
|  |  | (-0.94) | (-1.00) |
| *Health-related outcomes due to COVID-19* |  |  |  |
| Worsened health |  | 0.088 | 0.081 |
|  |  | (1.47) | (1.40) |
| Affected by COVID-19 |  | -0.017 | -0.008 |
|  |  | (-0.28) | (-0.13) |
| Felt sad/depressed more often |  | 0.023 | 0.028 |
|  |  | (0.35) | (0.43) |
| Felt anxious/nervous more often |  | 0.065 | 0.063 |
|  |  | (0.58) | (0.57) |
| Had trouble sleeping more often |  | -0.020 | -0.018 |
|  |  | (-0.30) | (-0.27) |
| Felt lonely more often |  | 0.059 | 0.057 |
|  |  | (0.95) | (0.92) |
| *Access to medical treatments* |  |  |  |
| Medical treatment cancelled by respondent |  | 0.193^***^ | 0.195^***^ |
|  |  | (5.73) | (5.84) |
| Medical treatment postponed/denied |  | 0.036 | 0.027 |
|  |  | (0.53) | (0.40) |
| Interview sample |  | -0.039 | -0.049 |
|  |  | (-0.51) | (-0.64) |
| *Context effects* |  |  |  |
| Confirmed deaths (per 100,000 inhabitants) |  |  | 0.269 |
|  |  |  | (1.72) |
| Duration of stay at home requirements |  |  | 0.298^**^ |
|  |  |  | (3.05) |
| $\sigma_{u_{0}}^{2}$ | 0.888^***^ | 0.849^***^ | 0.607^**^ |
|  | (3.61) | (3.46) | (2.89) |
| N (respondents) | 3092 | 3092 | 3092 |
| N (countries) | 27 | 27 | 27 |
| Deviance | 2978.1 | 2855.2 | 2842.9 |
| X^2^ | 280.6*** | 122.9*** | 135.3*** |

Data: SHARE Wave 8 COVID-19 Survey 1, Release version: 0.0.1 beta and SHARE Wave 8, Release version: 0 (weighted).
Entries are logistic regression coefficients with t-statistics in parentheses. $\sigma_{u_{0}}^{2}$ is not rescaled for models with explanatory variables.
Significance level: *: p<.05, **: p<.01, ***: p<.001.
